#  From lab-based studies to eye-tracking in virtual and real worlds: conceptual and methodological problems and solutions. Symposium 4 at the 20th European Conference on Eye Movement Research (ECEM) in Alicante, 20.8.2019. 

**DOI:** 10.16910/jemr.12.7.8

**Published:** 2019-11-25

**Authors:** Ignace T.C Hooge, Roy S. Hessels, Diederick C Niehorster, Gabriel J. Diaz, Andrew T. Duchowski, Jeff B. Pelz

**Affiliations:** Experimental Psychology, Helmholtz Institute, Utrecht University, Utrecht, The Netherlands; Utrecht University, The Netherlands; Lund University Humanities Lab, Lund University, Lund, Sweden; Rochester Institute of Technology, Rochester NY, USA; Visual Computing, Clemson University, USA; Rochester Institute of Technology, Rochester NY, USA

**Keywords:** fixations, saccades, data quality, face recognition

## Abstract

Wearable mobile eye trackers have great potential as they allow the measurement of eye movements during daily activities such as driving, navigating the world and doing groceries. Although mobile eye trackers have been around for some time, developing and operating these eye trackers was generally a highly technical affair. As such, mobile eye-tracking research was not feasible for most labs. Nowadays, many mobile eye trackers are available from eye-tracking manufacturers (e.g. Tobii, Pupil labs, SMI, Ergoneers) and various implementations in virtual/augmented reality have recently been released.The wide availability has caused the number of publications using a mobile eye tracker to increase quickly. Mobile eye tracking is now applied in vision science, educational science, developmental psychology, marketing research (using virtual and real supermarkets), clinical psychology, usability, architecture, medicine, and more. Yet, transitioning from lab-based studies where eye trackers are fixed to the world to studies where eye trackers are fixed to the head presents researchers with a number of problems. These problems range from the conceptual frameworks used in world-fixed and head-fixed eye tracking and how they relate to each other, to the lack of data quality comparisons and field tests of the different mobile eye trackers and how the gaze signal can be classified or mapped to the visual stimulus. Such problems need to be addressed in order to understand how world-fixed and head-fixed eye-tracking research can be compared and to understand the full potential and limits of what mobile eye-tracking can deliver. In this symposium, we bring together presenting researchers from five different institutions (Lund University, Utrecht University, Clemson University, Birkbeck University of London and Rochester Institute of Technology) addressing problems and innovative solutions across the entire breadth of mobile eye-tracking research.

Hooge, presenting Hessels et al. paper, focus on the definitions of fixations and saccades held by researchers in the eyemovement field and argue how they need to be clarified in order to allow comparisons between world-fixed and head-fixed eye-tracking research. - Diaz et al. introduce machine-learning techniques for classifying the gaze signal in mobile eye-tracking contexts where head and body are unrestrained. Niehorster et al. compare data quality of mobile eye trackers during natural behavior and discuss the application range of these eye trackers. Duchowski et al. introduce a method for automatically mapping gaze to faces using computer vision techniques. Pelz et al. employ state-of-the-art techniques to map fixations to objects of interest in the scene video and align grasp and eye-movement data in the same reference frame to investigate the guidance of eye movements during manual interaction.

**Video stream:** https://vimeo.com/357473408

